# Effects of *Bifidobacterium animalis* subsp. *lactis* IU100 on Immunomodulation and Gut Microbiota in Immunosuppressed Mice

**DOI:** 10.3390/microorganisms12030493

**Published:** 2024-02-29

**Authors:** Limian Zhou, Xindi Yin, Bing Fang, Jingjing He, Jing Zhan, Xiaoxu Zhang, Ran Wang

**Affiliations:** 1 Key Laboratory of Functional Dairy, Department of Nutrition and Health, China Agricultural University, Beijing 100190, China; 2022030083@mail.hfut.edu.cn (L.Z.);; 2 School of Food and Biological Engineering, Hefei University of Technology, Hefei 230601, China

**Keywords:** *Bifidobacterium animalis* subsp. *lactis* IU100, cyclophosphamide, gut microbiota, immunosuppression, immunomodulation, probiotic

## Abstract

Probiotics are live microorganisms with immunomodulatory effects in a strain-specific and dose-dependent manner. *Bifidobacterium animalis* subsp. *lactis* IU100 is a new probiotic strain isolated from healthy adults. This study aimed to evaluate the effects of IU100 on cyclophosphamide (CTX)-induced immunosuppression in mice. The results showed that IU100 significantly ameliorated CTX-induced decreases in body weight and immune organ indices. The promoted delayed-type hypersensitivity, serum hemolysins and immunoglobulin (IgA, IgG and IgM) levels after IU100 treatment indicated its enhancing role in cellular and humoral immunity. In addition, oral administration of IU100 increased serum cytokine (IL-1β, IL-2, IL-4, IL-6, IFN-γ, TNF-α) levels dose-dependently, which are associated with CTX-induced shifts in the Th1/Th2 balance. The probiotic IU100 also modulated the composition of gut microbiota by reducing the Firmicutes/Bacteroidetes ratio; increasing beneficial *Muribaculaceae* and the *Lachnospiraceae* NK4A136 group; and inhibiting harmful *Clostridium sensu stricto* 1, *Faecalibaculum* and *Staphylococcus* at the genus level. The above genera were found to be correlated with serum cytokines and antibody levels. These findings suggest that IU100 effectively enhances the immune function of immunosuppressed mice, induced by CTX, by regulating gut microbiota.

## 1. Introduction

The immune system is vital in defending the host from external viruses, pathogenic bacteria, parasites, fungi, yeasts and internal abnormal cells like tumor cells [1]. Immune disorders, including the underactivity and overactivity of immune function, are linked with the dysregulation of multiple immune organs, cells and molecules [2]. In recent years, the bidirectional communication between host immunity and gut microbiota has been recognized. Many immune disorders, such as inflammatory and autoimmune diseases, are associated with gut dysbiosis [3]. Meanwhile, the innate and adaptive immune systems participate in shaping microbial composition [4]. A better understanding of the immune system and microbiota crosstalk is essential in developing bacteria-targeting therapeutic approaches for immune-mediated diseases.

Cyclophosphamide (CTX) is a widely used alkylated drug for the chemotherapy of cancers such as leukemia, breast cancer, lymphoma and autoimmune diseases [5]. Its primary mechanism of action is an antimitotic effect through the alkylation of DNA, which results in cell death [6]. However, the cytotoxic effects of CTX on tumor cells are not specific; CTX also exerts myelosuppression and immunosuppression [7,8]. Furthermore, CTX causes severe intestinal problems, such as gut microbiota dysbiosis, mucosal injury and disrupted barrier function [9,10]. It is essential to combine CTX treatment with effective supplements to minimize side effects and improve anticancer efficacy.

Many studies have reported that probiotics exhibit immunity-enhancing effects in CTX-treated mice. A multispecies combination of *Lactobacillus* and *Bifidobacterium* probiotics improves the immune organ indices, immune cell counts, cytokine levels, intestinal morphology and the gut microbiota [11]. *Lactobacillus plantarum* NCU116 has been shown to regulate Th17/Treg cell immune responses, reinforce the intestinal barrier, increase microbial diversity and increase fecal short-chain fatty acid (SCFA) levels [10,12]. It is recognized that the major roles of probiotics in the regulation of immune function are mediated with bacteria cell components or metabolites via interplay with intestinal epithelial cells (IECs) or immune cells through surface receptors, thereby stimulating different signal pathways and activating the systemic immune system [13,14,15]. Moreover, probiotics are usually associated with the alteration of gut microbiota in the host [16]. They contribute to maintaining intestinal homeostasis by inhibiting harmful microorganisms’ growth [17]. Taken together, probiotics have great potential as natural immunomodulatory agents against impaired immunity.

*Bifidobacterium animalis* subsp. *lactis* IU100 (IU100) is a probiotic strain isolated from the feces of healthy adults. It displays several bioactivities in vitro and in vivo [18,19]. Currently, the regulation effect of IU100 on gut microbial composition and immune function is unknown. Herein, the effects of IU100 on body weight; the thymus and spleen indices; immune organ histopathology; delayed-type hypersensitivity; serum hemolysins; the levels of cytokines and immunoglobulins; and gut microbiota composition were investigated in CTX-induced immunosuppressed mice.

## 2. Materials and Methods

### 2.1. Bacterial Culture and Preparation

*Bifidobacterium animalis* subsp. *lactis* IU100 (CGMCC No. 12942) was obtained from the China General Microbiological Culture Collection (CGMCC, Beijing, China). IU100 was cultured in MRSc medium (De Man, Rogosa and Sharpe with 0.05% (*w*/*v*) L-cysteine) at 37 °C for 24 h. Bacterial cell counts were assessed using the plate count method before centrifugation (6000× *g* for 10 min) and washing. The harvested IU100 cells and *Bifidobacterium animalis* subsp. *lactis* BB-12 (BB12) lyophilized powder (Chr. Hansen Holding A/S, Hoersholm, Denmark) were suspended in sterile saline and diluted for the following experiment.

### 2.2. Animals

Sixty male specific pathogen-free (SPF) BALB/c mice (body weight 21.99 ± 0.14 g) at an age of 4 weeks were purchased (Vital River Laboratories, Beijing, China) and raised in a standard environment (22 °C ± 2 °C) with a 12 h light/dark cycle. Animals had *ad libitum* access to water and diet. The animal management and experimental protocols were conducted with the approval of the Institutional Animal Care and Use Committee, China Agricultural University, Beijing, China (Approval No. AW21402202-5-2).

### 2.3. Experimental Design

**Figure 1:**
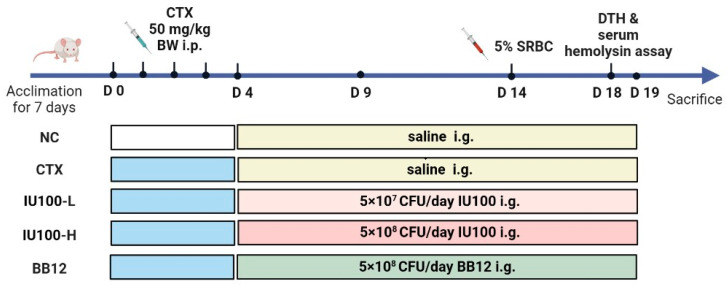
Experimental protocol of this study. NC: normal control group; CTX: CTX-treated model group; IU100-L: low-dose IU100 group; IU100-H: high-dose IU100 group; BB12: positive control BB12 group. i.p., intraperitoneal injection. i.g., intragastric administration. BW, body weight.

After adaptation, mice were assigned to 5 groups: normal control group (NC), CTX-treated model group (CTX), CTX-treated with low-dose IU100 group (IU100-L, 5 × 10^7^ CFU/day), CTX-treated with high-dose IU100 group (IU100-H, 5 × 10^8^ CFU/day), CTX-treated with BB12 (BB12, 5 × 10^8^ CFU/day). The immunosuppression was induced by 50 mg/kg of CTX via intraperitoneal injection for 5 consecutive days, as depicted in Figure 1. Mice in the NC group received an injection of sterile saline. From day 5 onward, the NC and CTX groups received saline intragastrically; the other three groups received the same number of different probiotics for 15 days. Mouse body weights were measured during the whole period. On day 20, mice were anesthetized with diethyl ether after 12 h of fasting. Blood, spleen, thymus and feces samples were collected and preserved for further analysis.

### 2.4. Determination of Thymus and Spleen Indices

The spleen and thymus were immediately removed and weighed after the mice were sacrificed. The spleen index and thymus index (mg/g) were calculated as the ratio of thymus (or spleen) weight to body weight [20].

### 2.5. Histological Analysis

The sections of the spleen and thymus were prepared and scored as previously reported [21]. Briefly, after fixing in 4% paraformaldehyde solution and embedding in paraffin, samples were sliced into 4 μm thickness sections and stained with hematoxylin and eosin (HE) (Service Bio Co., Wuhan, China). The images were observed with a light microscope (200× magnification, Leica, Wetzlar, Germany).

A semi-quantitative scale of histopathologic thymic scores was based on the degree of cortex-to-medullary (C/M) boundaries and lymphocytic depletion: 0 (distinct C/M boundaries, no lymphocytic depletion), 1 (distinct C/M boundaries, mild lymphocytic depletion), 2 (slightly indistinct C/M boundaries, moderate lymphocytic depletion) and 3 (indistinct C/M boundaries, marked lymphocytic depletion). A semi-quantitative scale of splenic scores was based on the degree of lymphocytic depletion: 0 (none), 1 (minimal), 2 (mild), 3 (moderate) and 4 (severe).

### 2.6. Delayed-Type Hypersensitivity in Mice (DTH)

DTH was assessed according to a previously described method [22]. Eight mice of each group were intraperitoneally injected with 0.2 mL of 5% sheep red blood cell (SRBC) on day 14. The baseline of the left rear footpad thickness in the mice was measured. After four days, mice were given a subcutaneous injection of 0.02 mL of 20% SRBC in the left rear footpad, and thickness was recorded after 24 h. The differences in thicknesses were evaluated as DTH.

### 2.7. Serum Hemolysin Assay

For the serum hemolysin assay, mice received 0.2 mL of 5% SRBC via intraperitoneal injection. Then, peripheral blood was collected from the eye orbit after 4 days and centrifuged for serum. The serum was diluted 100 times with sterile saline for the following serum hemolysin assay, as previously described [9].

### 2.8. Determination of Serum Cytokine and Immunoglobulin Contents

The serum cytokines (IL-2, IL-4, IL-6, IL-1β, TNF-α and IFN-γ) and immunoglobulins (IgA, IgM and IgG) were determined via enzyme-linked immunosorbent assay (ELISA) using commercial kits (Sinogene Biotech Co., Ltd., Beijing, China) in accordance with the manufacturer’s protocols.

### 2.9. Gut Microbiota Analysis

The genomic DNA in the fecal samples was isolated using the MOBIO Power Fecal DNA Isolation Kit (Qiagen, Stockach, Germany). The V3-V4 region of bacterial 16S rRNA was amplified by PCR using 338F and 806R universal primers [23]. PCR products were purified and quantified with the QuantiFluor-ST™ Blue Fluor Quantitative System. The amplicons were pooled for DNA library construction. The Illumina Novaseq6000 platform (Illumina, San Diego, CA, USA) was applied for sequencing.

The raw data were filtered with fastp (version 0.20.0) and merged using FLASH (version 1.2.11). Operational taxonomic units (OTUs) ≥ 97% similarity were clustered using UPARSE (version 7.1). The QIIME (version 1.9.0) software was used to analyze the diversity index and taxonomic structure of the microbial community. The correlations between the key bacteria and immunity-related parameters were analyzed with Pearson correlation. Linear discriminant analysis effect size (LEfSe) analyses were conducted to identify the differential abundance of taxa.

### 2.10. Statistical Analysis

Statistical analysis was performed with one-way analysis of variance (ANOVA) with Duncan’s multiple range test to compare differences between groups using SPSS (version 25.0, IBM Corp., Armonk, NY, USA). Figures were plotted with GraphPad Software Prism 8.0 (GraphPad Software Inc., Boston, MA, USA). Data are presented as mean ± SEM. *p* < 0.05 was considered statistically significant.

## 3. Results

### 3.1. Effects of IU100 on Body Weights, Immune Organ Index and Histopathology in Mice

**Figure 2:**
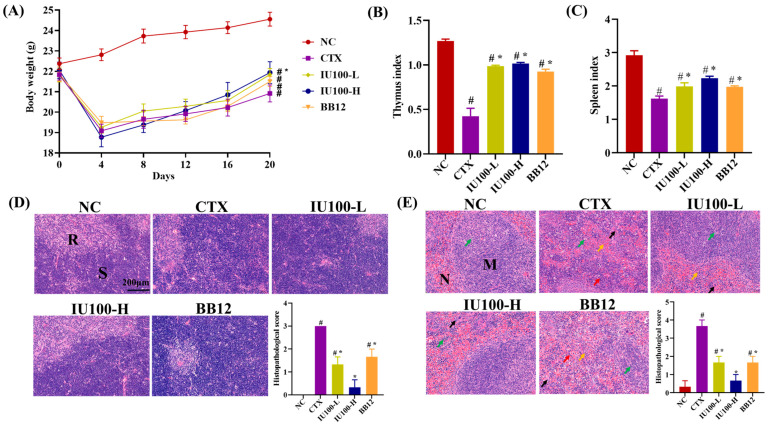
Effects of IU100 treatment on body weight, immune organ index and histopathology in CTX-treated mice. (**A**) Body weight. (**B**) Thymus index. (**C**) Spleen index. Histological examination and semi-quantitative scores of thymic (**D**) and splenic (**E**) sections (200× magnification). Data are expressed as the mean ± SEM, n = 12. # *p* < 0.05 vs. normal control group; * *p* < 0.05 vs. the CTX-induced model group. R: thymus medulla, S: thymus cortex, M: white pulp region of spleen, N: red pulp region of spleen. “↑” necrotic cell debris; “↑” red lineage cells; “↑” cranulocytes; “↑” megakaryocyte.

The body weights of mice in the NC group steadily increased during the experiment (Figure 2A). The injection of CTX induced a significant decrease (*p* < 0.05) in body weight and thymus/spleen indices in the mice. After intervention, mice in the IU100-H group had higher (*p* < 0.05) body weight than those in the model group. Oral administration of IU100 and BB12 significantly improved (*p* < 0.05) the thymus and spleen indices (Figure 2B,C). Histopathological observation and the scores of the thymus and spleen are shown in Figure 2D,E. The thymuses of CTX-treated mice showed thymic medulla atrophy and relatively enlarged cortical areas compared with the NC group. When treated with a high dose of IU100, thymic lobules are structurally normal without obvious atrophy. The cortex and medulla were divided clearly. Mice in the NC group had thicker spleen capsules. The spleen parenchyma of mice in the CTX group were arranged disorderly. The CTX-treated mice had scattered white pulp and germinal centers. After the IU100 treatment, the mice had improved morphology manifested by relatively complete spleen capsules, larger white marrow and a closely arranged germinal center. In addition, the histopathologic scores of the thymus and spleen after the probiotic IU100 treatment were significantly lower (*p* < 0.05) than those in the CTX group. These results demonstrate that IU100 ameliorated the spleen and thymus injuries induced by CTX in mice.

### 3.2. Effects of IU100 on DTH Reactions and Serum Hemolysin in Mice

**Figure 3:**
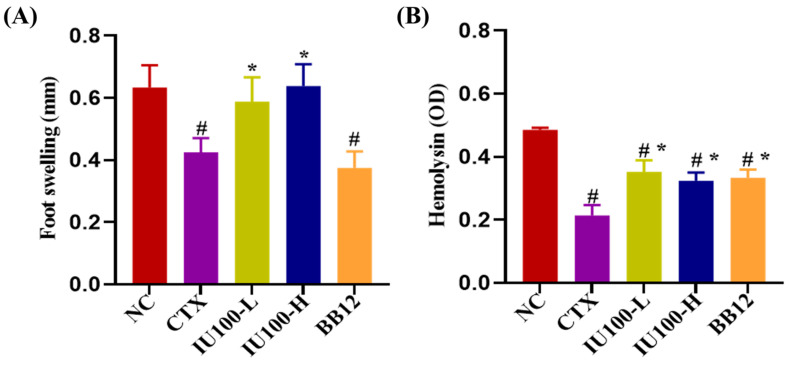
Effects of IU100 on SRBC-induced DTH (**A**) and serum hemolysin levels (**B**) in CTX-treated mice. Data are expressed as the mean ± SEM, n = 8. # *p* < 0.05 vs. normal control group; * *p* < 0.05 vs. the CTX-induced model group.

To understand the regulation effect of IU100 on cellular immune function, the DTH response was used to analyze the degree of tissue turgor. In Figure 3A, foot swelling was significantly reduced (*p* < 0.05) in the CTX group compared with the NC group. In contrast, both low and high doses of the IU100 treatment significantly improved (*p* < 0.05) foot edema, whereas BB12 administration had no effect on DTH reactions.

To determine the regulation of IU100 on humoral immune function, serum hemolysin levels were measured after immunization, as shown in Figure 3B. The serum hemolysin levels in the CTX group were significantly lower (*p* < 0.05) than those in the NC group. The probiotic IU100 and BB12 supplements all significantly increased (*p* < 0.05) serum hemolysin levels.

### 3.3. Effects of IU100 on Serum Cytokine and Immunoglobulin Levels in Mice

**Figure 4:**
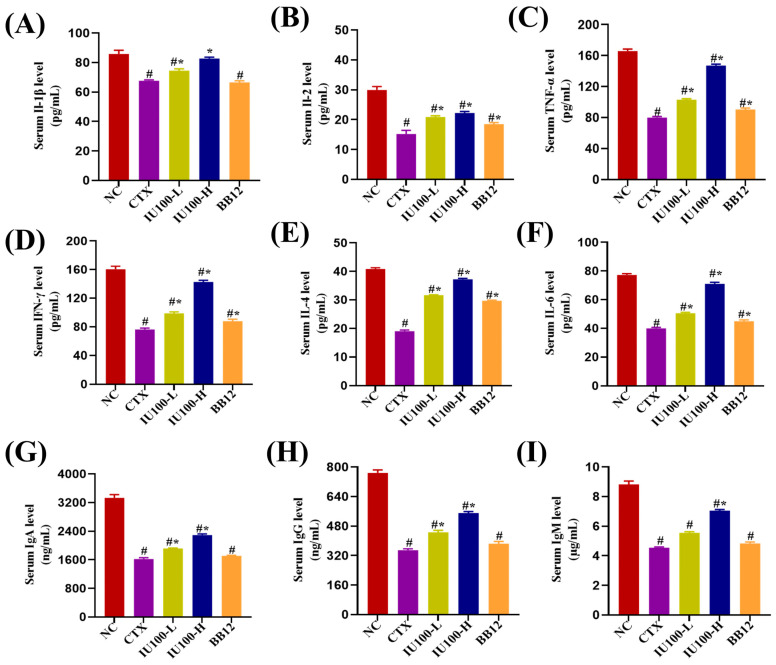
Effects of IU100 on serum cytokines (**A**) IL-1β, (**B**) IL-2, (**C**) TNF-α, (**D**) IFN-γ, (**E**) IL-4 and (**F**) IL-6 and serum IgA (**G**), IgG (**H**) and IgM (**I**) levels in CTX-treated mice. Data are expressed as the mean ± SEM, n = 12. # *p* < 0.05 vs. normal control group; * *p* < 0.05 vs. the CTX-induced model group.

As shown in Figure 4A–F, serum IL-1β, IL-2, TNF-α, IFN-γ, IL-4 and IL-6 levels were decreased (*p* < 0.05) in the CTX group compared with the NC group. Mice in the BB12 group had higher (*p* < 0.05) IL-2, TNF-α, IFN-γ, IL-4 and IL-6 levels compared with the CTX group. After the oral administration of IU100, IL-1β, IL-2, TNF-α, IFN-γ, IL-4 and IL-6 levels were restored (*p* < 0.05) in a significantly dose-dependent manner, suggesting that IU100 is an immunomodulatory probiotic that regulates cytokine secretion.

In Figure 4G–I, mice in the CTX group had significantly reduced (*p* < 0.05) serum IgA, IgG and IgM levels than those in the NC group. The levels of IgA, IgG and IgM in mice with IU100 administration increased (*p* < 0.05) compared with the CTX group. Meanwhile, immunoglobulins were higher in the IU100H group than in the IU100-L group. The BB12 administration had no effect on serum immunoglobulin levels. These results highlighted that IU100 can reduce the immunosuppressive effect of CTX in mice by enhancing humoral immunity.

### 3.4. Effects of IU100 on the Gut Microbiota

**Figure 5:**
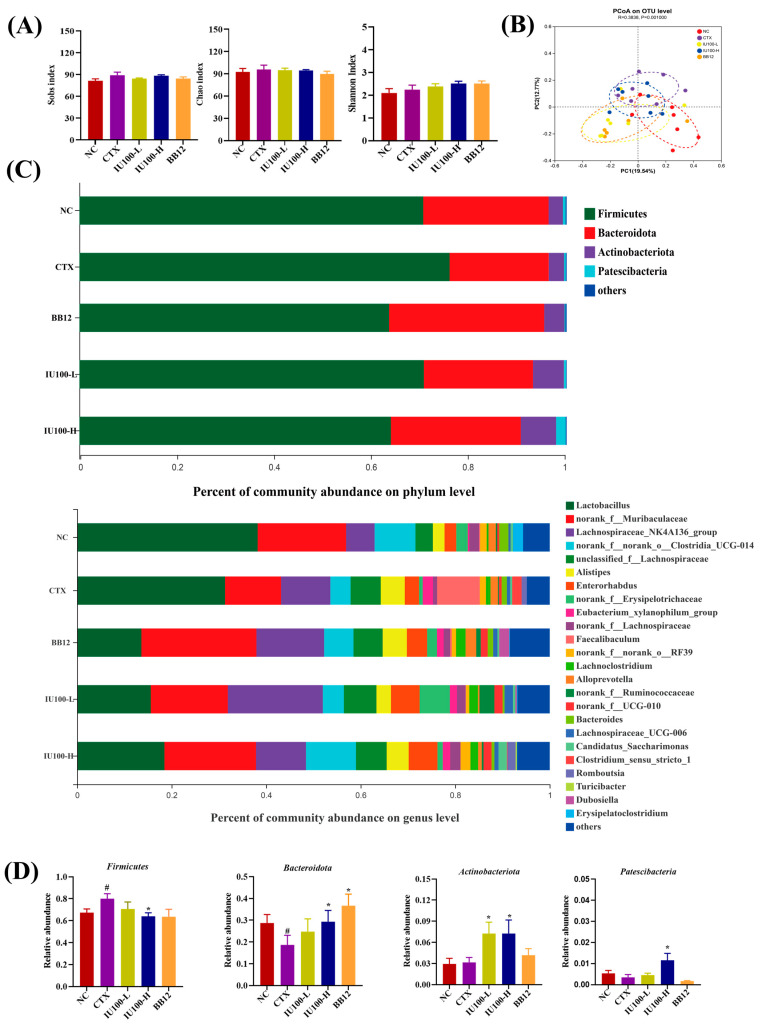
The effects of IU100 on the structure and composition of the gut microbiota. (**A**) Bacterial richness and diversity estimated by the Sobs, Chao and Shannon indices at the phylum level. (**B**) Bacterial β-diversity assessed by PCoA based on the unweighted UniFrac distances. (**C**) Gut microbiota composition at the phylum and genus levels. (**D**) Comparison of relative abundance of dominant phyla. Data are expressed as the mean ± SEM, n = 7. # *p* < 0.05 vs. normal control group; * *p* < 0.05 vs. the CTX-induced model group.

To evaluate the effects of IU100 on the gut microbiota in immunocompromised mice, fecal samples were analyzed via 16S rRNA sequencing. There were no significant differences in the Sobs, Chao or Shannon indices between groups (Figure 5A). The PCoA results demonstrated that the structure of the microbial community in each group was different (*p* = 0.001, Figure 5B). The PCoA displayed obvious separation between the groups; moreover, the gut microbiota of mice in the IU100L group clustered at a greater distance from the CTX group and relatively closer to the NC group.

The composition of the gut microbiota is shown in Figure 5C,D. The dominant phyla were Firmicutes, Bacteroidota, Actinobacteria and Patescibacteria, whose respective proportions varied in different groups, accounting for over 98% of the total fecal bacterial community. Compared with the NC group, the abundance of Firmicutes in the CTX group increased (*p* < 0.05), while the abundance of Bacteroidota decreased (*p* < 0.05). After the IU100 intervention, the abundance of Firmicutes decreased (*p* < 0.05) in the IU100-H group, and the abundance of Bacteroidota increased (*p* < 0.05) in both the IU100-L and IU100-H groups. In particular, the abundance of Actinobacteria and Patescibacteria increased (*p* < 0.05) after IU100 administration.

Down to the genus level, CTX induced a higher abundance of *Faecalibaculum*, *Clostridium*_*sensu*_*stricto*_1 and *Alloprevotella* and a lower abundance of *Romboutsia*, *Erysipelotrichaceae*, *Lachnoclostridium*, *Ruminococcaceae* and *Lactobacillus*. Compared with the CTX group, IU100 increased the *Muribaculaceae*, the *Lachnospiraceae* NK4A136 group, *Enterorhabdus*, *Erysipelotrichaceae*, *Lachnoclostridium*, *Ruminococcaceae*, *Lachnospiraceae* UCG-006, *Candidatus Saccharimonas*, *Turicibacter*, *Dubosiella* and *Romboutsia* and decreased the abundance of *Faecalibaculum*, *Alistipes*, *Alloprevotella*, *Bacteroides* and *Clostridium*_*sensu*_*stricto*_1.

**Figure 6:**
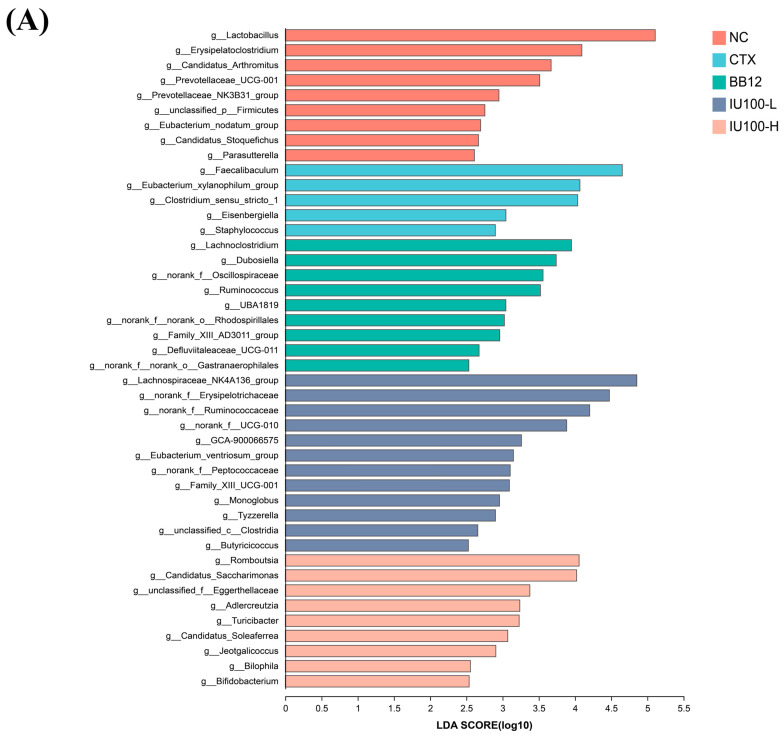

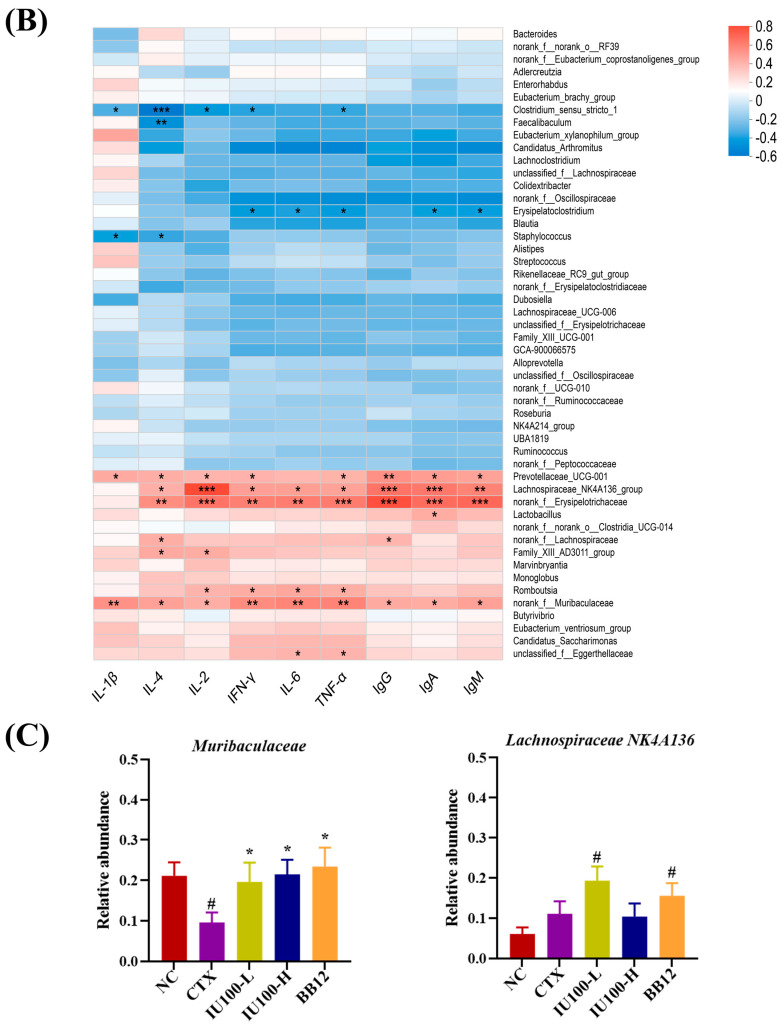
LEfSe analyses of the fecal microbiota in different groups (**A**) and Pearson correlation analysis between 50 dominant bacterial genera and immune parameters (**B**). (**C**) Relative abundance of the top 2 most abundant bacteria with significant differences between groups. # *p* < 0.05 vs. normal control group; * *p* < 0.05, ** *p* < 0.01, *** *p* < 0.001 vs. the CTX-induced model group.

LEfSe analysis was performed, and linear discriminant analysis (LDA > 2.5) was used to evaluate the effect size of taxa with significant differences. In Figure 6A, a total of 44 different taxa at the genus level were identified. This showed that CTX mainly enriched the abundance of *Faecalibaculum*, the *Eubacterium xylanophilum* group, *Clostridium*_*sensu*_*stricto*_1, *Eisenbergiella* and *Staphylococcus*. Compared with the CTX group, IU100-L increased the abundance of the *Lachnospiraceae* NK4A136 group, *Erysipelotrichaceae*, *Ruminococcaceae*, UCG-010, GCA-900066575, the *Eubacterium ventriosum* group, *Peptococcaceae*, Family XIII UCG-001, *Monoglobus*, *Tyzzerella*, *Clostridia* and *Butyricicoccus*. In the IU100-H group, the abundance of *Romboutsia*, *Candidatus Saccharimonas*, *Eggerthellaceae*, *Adlercreutzia*, *Turicibacter*, *Candidatus Soleaferrea*, *Jeotgalicoccus*, *Bilophila* and *Bifidobacterium* was greatly enhanced.

The correlations between the core bacteria and the host immune-related parameters were identified using Pearson correlation analysis. As depicted in Figure 6B, CTX-enriched *Clostridium*_*sensu*_*stricto*_1, *Faecalibaculum* and *Staphylococcus* had negative correlations (*p* < 0.05) with IL-1β, IL-4, IL-2, IFN-γ and TNF-α, while the IU100 treatment increased the key bacteria of the *Lachnospiraceae* NK4A136 group, *Erysipelotrichaceae*, *Muribaculaceae*, *Romboutsia* and *Eggerthellaceae* positively correlated (*p* < 0.05) with IL-1β, IL-2, IFN-γ, TNF-α, IL-4, IL-6, IgG, IgA and IgM. In addition, the top two most abundant genera with significant differences are presented in Figure 6C. After the IU100 intervention, the proportions of beneficial *Muribaculaceae* and the *Lachnospiraceae* NK4A136 group were higher (*p* < 0.05) in immunosuppressed mice. Overall, these results suggested that IU100 effectively influenced the CTX-altered gut microbiota structure and, therefore, played a critical role in immunomodulation.

## 4. Discussion

The potential of probiotics to mitigate the adverse effects of CTX-induced immunosuppression during anticancer treatment has gained increasing attention. In this study, the modulatory role of IU100 in non-specific, cell-mediated and humoral immunity was first explored in a CTX-induced immunocompromised mouse model. The results indicated that the combined treatment of IU100 and CTX resulted in the improvement of body weight; the thymus/spleen indices; pathological damage; the DTH response; serum hemolysin; cytokine (IL-1β, IL-2, IL-4, IL-6, TNF-α and IFN-γ) and immunoglobulin (IgA, IgG, IgM) production; and gut microbiota disorders. This evidence highlights the prospective application of IU100 as a dietary immunostimulating supplement.

IU100 is a Gram-positive lactic acid bacterium belonging to the Actinobacteria phylum and the *Bifidobacterium* genus. Previous studies have demonstrated that *Bifidobacterium* is one of the most well-studied probiotics with excellent immune-enhancing effects [24]. Research suggests that the main mechanisms by which it influences immune function include communication with IECs or immune cells in the lamina propria through pattern recognition receptors such as Toll-like receptors (TLRs); the induction of different cytokines or chemokines; and the maintenance of gut microbial homeostasis [14]. Many studies have explored the relationship between probiotics and intestinal cells. Complex probiotics comprising *Bifidobacterium animalis* subsp. *lactis* XLTG11 increase goblet cell counts regulating mucin synthesis and barrier function [11]. *Bifidobacterium breve* MCC-117 activates the TLR-2-mediated NF-κB pathway by upregulating A20, Bcl-3, Tollip and SIGIRR regulators in porcine intestinal epithelial cells [25]. The consumption of *Bifidobacterium breve* prevents intestinal inflammation through the induction of intestinal IL-10-producing regulatory T cell subset Tr1 cells [26]. These interactions lead to the activation of non-specific and specific immunity and the secretion of effector molecules such as cytokines and immunoglobulins. Moreover, probiotics stimulate macrophage functionality and effector molecule production distant from the intestine, such as in the peritoneum, spleen, bronchus and mammary glands [15]. The administration of *Bifidobacterium animalis* subsp. *lactis* Bb12 induces IL-10 secretion through the TLR-2 pathway in the cells of porcine mesenteric lymph nodes (MLN) [27]. *Bifidobacterium longum* BBMN68 has been shown to boost peritoneal macrophages’ phagocytic activities; CD11c+ and CD103+ dendritic cell (DC) induction in MLN; and splenic lymphocyte proliferation [28,29,30].

Herein, CTX decreased the thymus/spleen indices, serum cytokines (IL-1β, IL-2, IL-4, IL-6, IFN-γ and TNF-α), immunoglobulins (IgA, IgG and IgM), serum hemolysin, DTH responses and body weights in mice, which is consistent with previous studies [8,9,31]. The thymus and spleen are two important immune organs, and their indices indicate non-specific immunity [32]. Cytokines, small-molecular-weight messengers produced by various cells, play critical roles in complicated immune system networks, differentiation, the function of CD4+ T cells and hematopoiesis regulation. IL-2 is a growth factor for T cells [33]. TNF-α is a pro-inflammatory cytokine that helps initiate and propagate the inflammatory response and cancers; it also has anti-tumor effects through apoptosis or necrosis [34]. IFN-γ is a key regulator in the activation of cellular immunity [35]. IL-4 is crucial for the promotion of naive CD4+ T cell differentiation into Th2 cell antibody production [36]. The Th1-associated IL-2, IFN-γ and TNF-α and the Th2-associated IL-4 contribute to maintaining a dynamic Th1/Th2 balance in hosts. IL-1β stimulates the activation and proliferation of T/B cells [37]. IL-6 stimulates the differentiation and activation of T/B cells; it is also involved in the regulation of hematopoiesis [38]. IgA, IgG and IgM are synthesized by B cells, and their levels in the body reflect humoral immunity function. In addition, serum hemolysis and DTH are important indicators of humoral and cellular immune function, respectively [31,39]. Our data showed that IU100 effectively accelerated recovery from CTX-induced suppression in non-specific, cellular and humoral immune responses by upregulating the thymus/spleen indices, cytokine and immunoglobulin levels, serum hemolysis and DTH, eventually leading to the restoration of body weight.

CTX is a commonly used alkylating chemotherapeutic agent that has a broad spectrum of anticancer activities and immunosuppressive properties [40]. It usually causes gut bacterial dysbiosis, which causes disrupted barrier function and imbalanced mucosal immunity [41]. Accumulating data indicate that gut microbes are effective in maintaining gut homeostasis [42]. Herein, IU100 administration significantly moved the composition of the microbial community toward a healthy profile. Similar to previous reports, mice in the CTX group showed increased Firmicutes and decreased Bacteroidota at the phylum level and increased *Staphylococcus* and decreased *Muribaculaceae* at the genus level [9,11]. This phenomenon was reversed after the IU100 treatment. IU100 increased the phylum Bacteroidota and decreased Firmicutes. Furthermore, a Pearson correlation analysis revealed that multiple immune indicators, including cytokine and immunoglobulin levels, were positively correlated with *Muribaculaceae* and the *Lachnospiraceae* NK4A136 group. IU100 promoted the beneficial genera *Muribaculaceae* and the *Lachnospiraceae* NK4A136 group and suppressed harmful *Clostridium*_*sensu*_*stricto*_1, *Faecalibaculum* and *Staphylococcus*. It has been reported that *Muribaculaceae* abundance is strongly associated with propionate concentrations [43,44]. *Lachnospiraceae* NK4A136 group abundance is associated with health as a potential butyrate producer [45]. These SCFAs play multiple regulatory roles in the innate and adaptive immune systems [46]. Therefore, it is speculated that IU100 activated the immune system to secrete effector molecule cytokines and antibodies by lowering the Firmicutes/Bacteroidetes ratio and promoting bacteria that produced SCFAs.

This study not only provides evidence for the effects of probiotics on immune enhancement and CTX-induced side effect alleviation during chemotherapy but has also shed light on their important roles in tuning tumor fates. It has been suggested that there is a connection between the gut microbiota and cancer development and treatment responses [47]. Microbial taxa such as *Faecalibacterium*, *Bacteroides* and *Romboutsia* represent novel biomarkers in early tumor formation [48]. Probiotics have potential as alternative preventions and treatments of cancer by regulating tumor cell activity and the tumor microenvironment [49,50]. Therefore, the mechanism of probiotics affecting immune function by targeting specific bacteria and microbiota-derived metabolites is worth exploring in future studies.

## 5. Conclusions

In summary, this study proves that IU100 is protective against CTX-induced immunosuppression by inhibiting body weight loss and immune organ atrophy, as well as enhancing humoral and cellular immunity, in mice. Moreover, IU100 mitigated CTX-induced gut microbiota disorders by altering immune-associated core bacteria. These findings will contribute to a better understanding of the role of IU100 as an immunomodulatory agent and, therefore, provide a research basis for its further application.

## Data Availability

Data are contained within the article.
